# Global scenario of genetic diversity in *cox*1 and *nad*1 genes of *Moniezia expansa*

**DOI:** 10.1016/j.parepi.2023.e00333

**Published:** 2023-12-11

**Authors:** Ayed Alshammari, Umair Ali, Abdulbaset Mohammed Kabli, Majed H. Wakid, Muhammad Saqib, Shujaat Hussain, Warda Qamar, Mughees Aizaz Alvi

**Affiliations:** a Department of Biology, College of Science, University of Hafr Al Batin, Hafr Al Batin, Saudi Arabia; b Department of Clinical Medicine and Surgery, University of Agriculture, Faisalabad, Pakistan; c Department of Laboratory Medicine, Faculty of Applied Medical Sciences, Al-Baha University, Al-Baha, Saudi Arabia; d Department of Medical Laboratory Sciences, Faculty of Applied Medical Sciences, King Abdulaziz University, Jeddah 21589, Saudi Arabia; e Special Infectious Agents Unit, King Fahd Medical Research Center, Jeddah, Saudi Arabia; f Faculty of Veterinary and Animal Sciences, PMAS Arid Agriculture University, Rawalpindi, Pakistan; g Department of Parasitology, University of Agriculture, Faisalabad, Pakistan

**Keywords:** *Moniezia expansa*, *cox*1, *nad*1, Genetic diversity, Population structure, Phylogeny

## Abstract

Monieziasis is a parasite-borne production-limiting disease of livestock. *Moniezia expansa* is the most important species having cosmopolitan distribution. Despite of numerous prevalence reports, very little information is available about the evolutionary biology and population genetics of *M. expansa.* To close this research gap, this study was undertaken to recognize and inspect the genetic variation of *M. expansa* populations around the world using the *cox*1 and *nad*1 genes and deduce phylogenetic relationships with *M. expansa* populations. The *cox*1 and *nad*1 gene sequences were downloaded from the NCBI GenBank database. Followed by sequence alignment, median-joining networks were constructed using PopArt software. Diversity and neutrality indices were computed through DnaSp software while MEGA software was used to draw the maximum-likelihood phylogenetic tree. Thirty-two *cox*1 sequences, from five different countries, and 9 *nad*1 sequences from three different countries, were among the sequences used in this study. The *cox*1 and *nad*1 gene sequences had mutations in 97 and 36 different places, respectively. Twenty and 7 unique haplotypes were discovered for the *cox*1 and *nad*1 gene sequences, respectively. Comparable haplotype diversities were observed for both the genes under study (*cox*1 = 0.950; *nad*1 = 0.944). Negative Tajima's D and Fu Fs were found for the *cox*1 gene while these indices were positive for the *nad*1 gene. Phylogenetic analysis also showed the existence of unique haplotypes for both the *cox*1 and *nad*1 genes. The results of this study indicate that there is the existence of a huge genetic diversity in *M. expansa* isolates. For future studies, it is recommended that longer gene sequences should be used to describe genetic variation among *M. expansa* isolates as the length of the gene under study affects the genetic variation. Moreover, additional mitochondrial markers should also be investigated because the assertive strength of a group of gene targets is superior to defining genetic diversity.

## 1 Introduction

Monieziasis is a parasite-borne production-limiting disease of livestock (Hassanein et al., 2022; Nagarajan et al., 2022; Suleiman et al., 2022). The cestodes (tapeworms) of the genus *Moniezia* belong to the order Cyclophyllidea within the family Anoplocephalidae (Diop et al., 2015). *Moniezia* species are distributed throughout the world affecting livestock populations (Ghanim et al., 2022). The parasite uses oribatid mites as the intermediate host (Abdelhamid et al., 2021). Accidental ingestion of the infected mites by the definitive ruminant host leads to the release of larvae and attach themselves to host intestine and mature. After spending their whole lives in an animal's small intestine, tapeworms frequently leave the body (Guo, 2017).

In terms of prevalence, *Moniezia* species remain one of the most common helminthic parasites of ruminants in many ecological contexts (Diop et al., 2015). Although *Moniezia* tapeworms are typically thought to be mildly harmful, especially in adult livestock, gastrointestinal illnesses in calves and lambs can cause economic losses in stockbreeding (Mazyad and El-Nemr, 2002). Monieziasis has commercial potential because of the direct consequences it has on the host animals, which include diarrhea, intestinal obstruction, anemia, weight loss, weakness, decreased milk output, poor meat quality, and, in extreme cases, death (Hilegiworgise et al., 2019; Mafruchati, 2020). Perforation, intestinal blockage, perineal and hepatic abscesses, and cholecystitis are all caused by the parasite in sheep (Al-Otaibi et al., 2021).

So far, at least 12 *Moniezia* species have been described in domestic and wild ruminants based on their very narrow range of physical traits (Ohtori et al., 2015), which are sometimes convergent, causing dispute over the taxonomy of this genus (Diop et al., 2015). Of them, *M. expansa* and *M*. *benedeni* are the most frequently reported members of the genus *Moniezia*.

Mitochondrial DNA has been frequently used in studying intraspecifc variation in metazoans (Shen et al., 2010; Wei et al., 2010). Another approach for evaluating intraspecifc variation or genetic diversity in cestodes is microsatellite DNA, which has been shown to be very informative and is widely used in genetic population studies (Umhang et al., 2018). Despite its high prevalence, only a few papers on *Moniezia*'s genetic diversity are published (Diop et al., 2015; Guo, 2017).

Very little is known about their evolutionary biology and population genetics. Even though a few studies, notably those using partial *nad*1 and *cox*1 gene sequences, have discovered significant genetic variation within *M. expansa* reported from different parts of the world. The goal of this study was to recognize and inspect the genetic variation of *M. expansa* populations around the world using the *cox*1 and *nad*1 genes and deduce phylogenetic relationships with *M. expansa* populations.

## 2 Materials and methods

### 2.1 Data retrieval

A total of 41 gene sequences were collected after filtering the *M. expansa nad*1 (*n* = 9) and *cox*1 (*n* = 32) gene sequences available in the NCBI database.

### 2.2 Sequence alignment

All gene sequences were compiled into FASTA format using the MEGA software. Initially, all sequences were cut at both ends using the *cox*1 (MG099720) and *nad*1 (MG189623) reference sequences. Small sequences were removed, leaving 41 sequences for bioinformatics analysis, which included 354 bp *nad*1 (n = 9) and 527 bp *cox*1 (n = 32). The Maximum Likelihood technique and the Tamura-Nei model were used to infer the evolutionary history (Tamura et al., 2021).

### 2.3 Haplotype networking

The sequences were analyzed for haplotype analyses utilizing the DnaSP 6 tool (Rozas et al., 2017). The genetic composition of both gene areas was determined using diversity and neutrality indices. After converting the sequences to the file Nexus format, haplotype networking was done with the help of the PopArt program to provide a visual depiction of the haplotype linkages (Leigh and Bryant, 2015; Maddison et al., 1997).

## 3 Results

The NCBI database was used to retrieve 41 gene sequences of *M. expansa* for this study. Thirty-two *cox*1 sequences, from five different countries, and 9 *nad*1 sequences from three different countries, were among these sequences (Table 1).

**Table 1:** Accession numbers of gene fragments of *M. expansa* investigated in this study.

*cox*1			*nad*1		
Origin	No. of Isolates	Accession Numbers	Origin	No. of Isolates	Accession Numbers
Iraq	3	MH259793–94-95	Pakistan	1	OL963769
China	5	MG099720–21-22, NC036219, KX121041	Senegal	3	MG189626–27, LC102496
Senegal	15	AB821375–76–77-78-79-80/86–87–88-89-90-91-92-93, LC102496	China	5	MG189623–24-25, NC036219, KX121041
Ethiopia	8	AB821372–73-74/81–82–83-84-85			
India	1	OL689029			

### 3.1 Polymorphism and haplotype analysis

The *cox*1 gene sequences had mutations in 97 different places. The *nad*1 sequences had 36 different locations where mutations were discovered. After the investigation of 32 *cox*1 gene sequences, 20 unique haplotypes were discovered (Table 2). Hap15, the most prevalent haplotype among the six gene sequences, and three sequences each from Ethiopia and Senegal were present. The examination of 9 *nad*1 gene sequences revealed 7 unique haplotypes (Table 3). Hap06 and Hap07 served as the dominant haplotypes.

**Table 2:** Haplotypes of the *cox*1 gene of *Moniezia expansa*.

Name of haplotype	No. of Isolates	Accession Number
Hap01	1	MH259795-Iraq
Hap02	1	MH259794-Iraq
Hap03	1	MH259793-Iraq
Hap04	1	MG099722-China
Hap05	3	MG099721-China, NC036219-China, KX121041-China
Hap06	2	MG099720-China, LC102496-Senegal
Hap07	1	OL689029-India
Hap08	1	AB821393-Senegal
Hap09	1	AB821392-Senegal
Hap10	1	AB821391-Senegal
Hap11	1	AB821390-Senegal
Hap12	1	AB821389-Senegal
Hap13	3	AB821388-Senegal, AB821387-Senegal, AB821386-Senegal
Hap14	1	AB821385-Ethiopia
Hap15	6	AB821384-Ethiopia, AB821378-Senegal, AB821377-Senegal, AB821375-Senegal, AB821374-Ethiopia, AB821372-Ethiopia
Hap16	3	AB821383-Ethiopia, AB821382-Ethiopia, AB821381-Ethiopia
Hap17	1	AB821380-Senegal
Hap18	1	AB821379-Senegal
Hap19	1	AB821376-Senegal
Hap20	1	AB821373-Ethiopia

**Table 3:** Haplotypes of the *nad*1 sequences of *Moniezia expansa.*

Name Haplotype	No. of isolates	Accession numbers
Hap01	1	OL963769-Pakistan
Hap02	1	MG189627-Senegal
Hap03	1	MG189626-Senegal
Hap04	1	MG189625-China
Hap05	1	MG189624-China
Hap06	2	MG189623-China, LC102496-Senegal
Hap07	2	NC036219-China, KX121041-China

### 3.2 Haplotype network

**Fig. 1:**
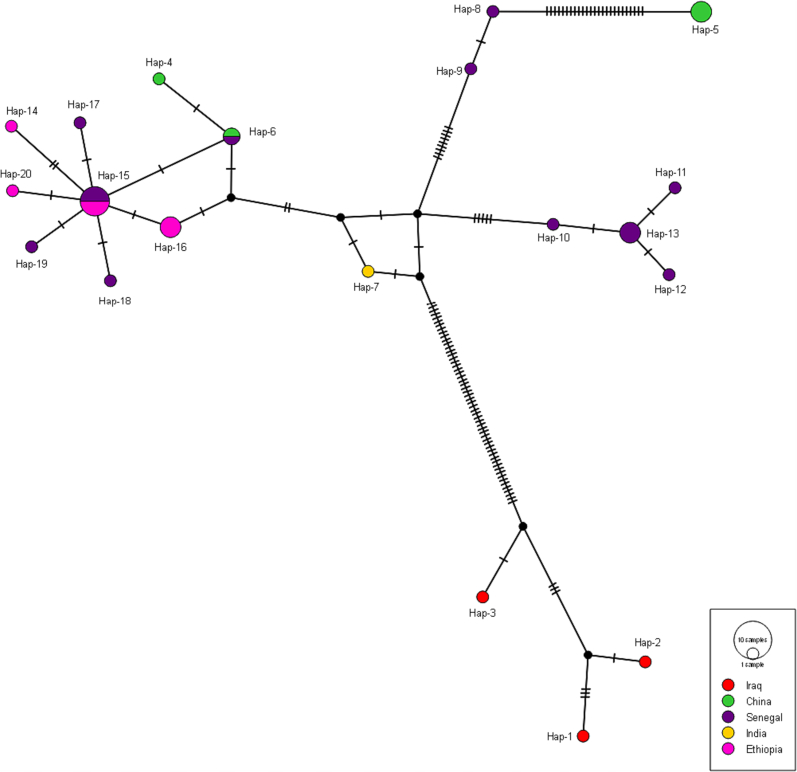
Median-joining network of the *cox*1 gene haplotypes of *Moniezia expansa*.

The *cox*1 network had 20 unique haplotypes (Table 2). According to the research, there were between one and 97 mutations (Fig. 1). Hap15, which made up 18.75% (6/32) of the network's total haplotypes, was the most common. With a combined 28.12% (9/32), Hap5, Hap13, and Hap16 placed second. The haplotypes in the network made up 46.87% (15/32) of all different single haplotypes. A single haplotype was provided by each of the following: Iraq (*n* = 3), Ethiopia (*n* = 2), Senegal (*n* = 8), India (*n* = 1), and China (n = 1).

**Fig. 2:**
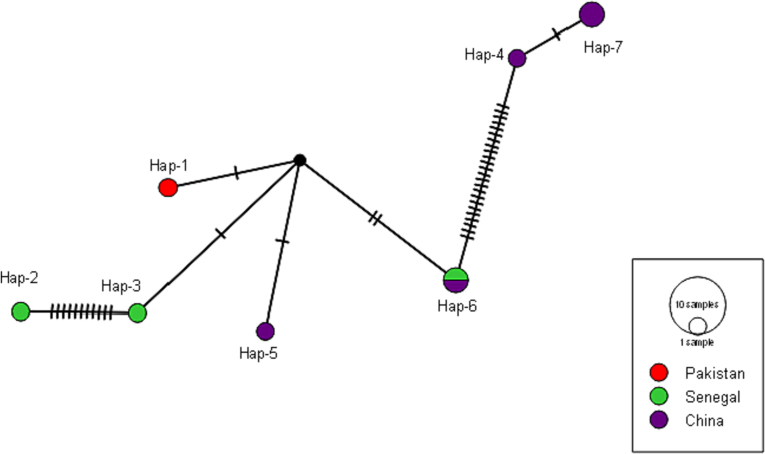
Median-joining network of the *nad*1 gene haplotypes of *Moniezia expansa*.

The *nad*1 haplotype network had 7 haplotypes (Table 3). When the primary haplotype was matched to the other haplotypes in this network, between one and twenty-five mutations were discovered (Fig. 2). The haplotype network was made up of 28.57% (2/7) of the most abundant haplotype, Hap06, and 28.57% (2/7) of Hap07. A single unique haplotype took up 71.42% (5/7) of the haplotype network. There was only one haplotype in Pakistan (n = 1), China (n = 2), and Senegal (n = 2).

### 3.3 Phylogenetic tree

**Fig. 3:**
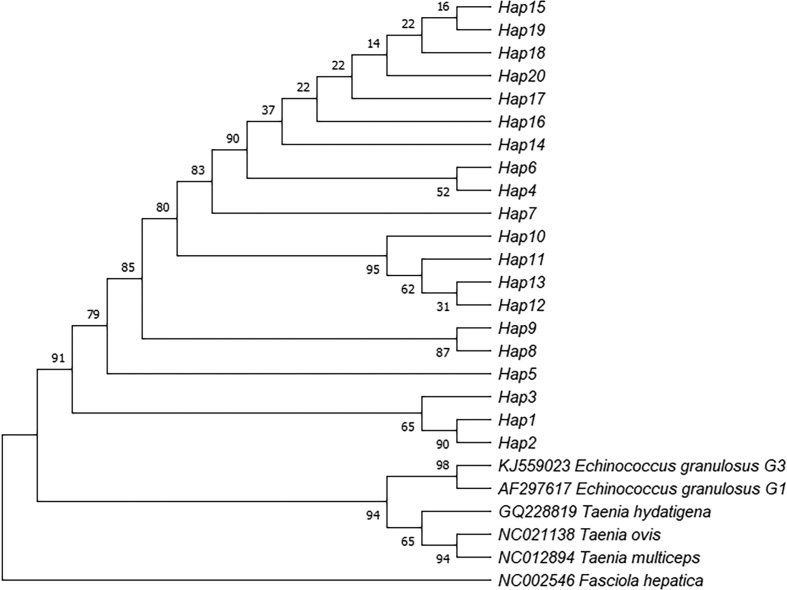
Maximum-likelihood phylogenic tree of *Moniezia expansa cox*1 gene.

**Fig. 4:**
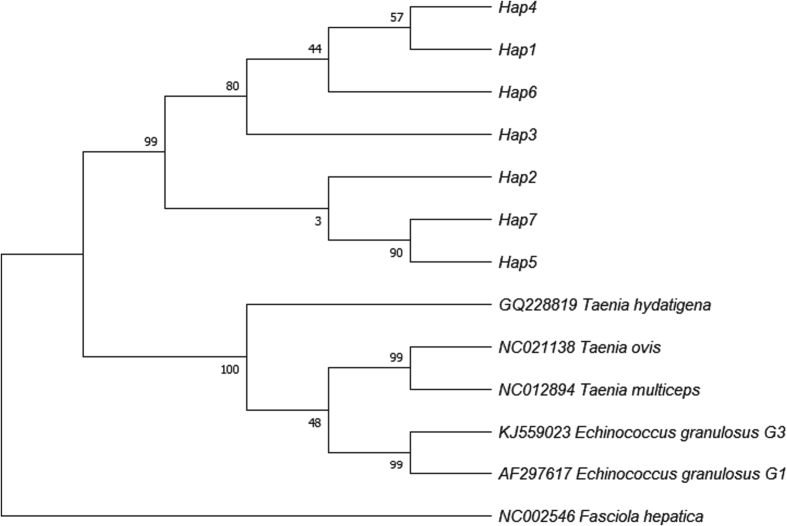
Maximum-likelihood phylogenic tree of *Moniezia expansa nad*1 gene.

The haplotype network's findings and those of the phylogenetic analysis agreed. As a result of aligning the *cox*1 and *nad*1genes sequences, phylogenetic trees were created, which are depicted in Fig. 3 and Fig. 4, respectively. *Fasciola hepatica* was used as an outgroup in both the trees. The trees with the highest log likelihood (*cox*1 = −7526.29; and *nad*1 = −4349.31) are shown.

### 3.4 Diversity analysis

The *cox*1 and *nad*1 groups' diversity and neutrality indices are shown in (Table 4). Negative values were found for Tajima D and Fu's Fs, for the *cox*1 gene indicating the existence of many alleles while the values of these indices were positive for the *nad*1 gene.

**Table 4:** Population indices of the *cox*1 and *nad*1 genes of *Moniezia expansa*.

Indices	*cox*1 (527 bp)	*nad*1 (354 bp)
No. of sequences	32	9
No. of mutations	97	36
Parsimony informative sites	80	27
No. of haplotypes	20	7
Haplotype diversity (Hd)	0.950 ± 0.024	0.944 ± 0.070
Nucleotide diversity (π)	0.03787 ± 0.00884	0.04402 ± 0.00781
Tajima's *D*	−0.64744	0.89114
Fu's Fs	−0.045	1.208
FLD	0.99968	0.63944
FLF	0.52414	0.78607

## 4 Discussion

Infectious diseases, especially parasitic infestations, are major health issues in both animals and humans, causing economic losses as well as severe illness (Alvi et al., 2022; Mo'awad et al., 2022; Rahman et al., 2020; Qamar et al., 2022). Parasites are responsible for diseases that result in significant economic losses due to lower productivity and illness (Babayani et al., 2022; Mahmoud et al., 2022). Infection with *Moniezia* species has significant risks for ruminants' productivity. A better understanding of genetic diversity is critical in a variety of fields of study, including epidemiology and molecular diagnostics (Cavallero et al., 2021).

Mitochondrial DNA has been utilized as a measure of population variety due to its high mutation rate. The *nad*1 and *cox*1 genes are extensively used DNA indicators in genetic research. Additionally, microsatellite DNA SSR markers have also been investigated for their ability to predict parasite population organization (Alberfkani et al., 2022; Gao et al., 2022; Palevich et al., 2020; Moendeg et al., 2017; Yin et al., 2016).

*Moniezia expansa* genetic diversity and population structure were investigated in this study. This was done using sequence information for the *nad*1 and *cox*1 gene sequences available in the NCBI Database. Gene sequences were downloaded from GenBank. Information on the global distribution of *M. expansa* infection's gene flow and population dynamics was revealed by the study's findings. To ascertain the genetic diversity and variations of the *M. expansa* isolates, we conducted in-silico analyses using 9 *nad*1 (354 bp) and 32 *cox*1 (527 bp) gene sequences.

*Moniezia* was not regarded by Elliott's studies as a possible cause of sheep diarrhea (Elliott, 1984). On the other hand, Cabaret et al. (2006) discovered a connection between lamb diarrhea and the prevalence of *Moniezia* (Cabaret et al., 2006). So, there is a need to assess how cestodes affect sheep health and productivity. *Moniezia* species infection is frequently treated with the benzimidazole anthelmintics. Helminth drug-metabolizing enzymes may modify the potency of anthelmintic treatment, resulting in anthelmintic resistance. Anthelmintic resistance in *Moniezia* has not been well-documented. However, Prchal et al. (2015) discovered that *M. expansa* is capable of deactivating the provided anthelmintics, protecting itself from their activity, which leads to anthelmintic resistance (Prchal et al., 2015). Targeted selective therapies are only applied to animals that have been diagnosed as unwell in order to decrease the usage of anthelmintics to avoid the development of anthelmintic resistance. This is based on the observation that small ruminant gastrointestinal parasite populations are heavily aggregated and disseminated throughout the flocks of these animals (Gaba et al., 2005).

A total of 20 *cox*1 haplotypes and 7 *nad*1 haplotypes were represented by the 41 samples that were examined and high genetic diversity among *M. expansa* was noted. In a previous study related to *E. granulosus*, another cestode, similar types of results were observed (171 haplotypes after analyzing 212 samples with Hd = 0.994) (Kinkar et al., 2018). The length of the gene under study is a major factor in the haplotype variations between researches. As a result, sequencing larger mitochondrial gene segments can identify more haplotypes. Neutrality measures were used to assess genetic variability and population expansion (Ramos-Onsins and Rozas, 2002). The positive value of Tajima's D shows heterozygosity, whereas the negative value of Tajima's D indicates that one allele has a selective advantage over the other. The Tajima's D test determines how far the population deviates from the classic neutral model. The negative result also suggests considerable population expansion (Stephens et al., 2001; Vamathevan et al., 2008). The Tajima's D value for the *nad*1 gene sequences was larger (0.89114) than that of the *cox*1 gene sequences (−0.64744). The neutrality index's negative value shows that this expansion may continue to climb in the coming years. Fu's Fs, with a significantly negative value (*p* < 0.05), works as a marker of population growth sensitivity, showing that the populations share the same gene pool and have similar growth predispositions. (Fu, 1997; Li et al., 2009). We found that both the *nad*1 and *cox*1 haplotype groups had low and statistically insignificant Fu's Fs values indicating the vulnerability of the study population to global expansion. The population polymorphism was measured using the computation of the nucleotide diversity. We discovered that the *nad*1 (0.04402) gene sequence had a greater mean nucleotide difference than the *cox*1 (0.03787) gene sequence. Haplotype diversity was also assessed to establish the distinctiveness of haplotypes within the study population. In our study, the *nad*1 (0.944) and *cox*1 (0.950) gene sequences had similar values. We examined the *cox*1 gene sequences and detected 20 different haplotypes. The network was divided into 20 unique haplotypes. The analysis of the *nad*1 gene sequences revealed seven different haplotypes. Five unique haplotypes with the major haplotype accounting for 71.42% of the overall network were identified. A single ancestor is represented by the major haplotypes. The 527 bp *cox*1 gene sequences had a total of 97 different mutations, whereas the 354 bp *nad*1 sequences contained 36 different mutations. The greater mutation rates may reflect *M. expansa* longer and more convoluted evolutionary history. The genetic diversity of *M. expansa* is quite high throughout the world, and the complex phylogeographic patterns revealed by phylogenetic and topographical analyses suggest that the rigorous animal trade has had a significant impact on the species' current distribution, as seen for *E. granulosus*. (Kinkar et al., 2018). As *Moniezia* is a neglected parasite, very few molecular investigations, even fewer on population structure, have been carried out during the past few years. Thus, we were unable to compare the results of the current study with other molecular studies encompassing *Moniezia* species.

## 5 Conclusions and recommendations

Information on the population structure of *M. expansa* is scarce. To close this research gap, we chose *nad*1 and *cox*1 gene sequences from GenBank in this work since these two genes are often used to evaluate genetic diversity. To describe genetic diversity, 527 base pair *cox*1 and 354 base pair *nad*1 gene consensus sequences were analyzed in this study. The results of this study indicate that there is high genetic diversity in *M. expansa* isolates as depicted by the median-joining network, phylogenetic tree, and population indices. Longer, rather than shorter, gene sequences should be used in the future to describe genetic variation among *M. expansa* isolates, as the previous investigations into genetic variation have been shown to be influenced by the length of the gene under investigation. It is also recommended that additional mitochondrial markers be investigated because the assertive strength of a group of genes is superior to defining genetic diversity.

## Authors' contributions

The study was conceptualized by MAA, AA, and UA. MAA, AMK, and MHW created the methodology, while MAA, SH, and MS implemented it. MAA and UA prepared the original draft, while WQ and MHW handled the review and editing. The submitted version of the manuscript has been read and approved by all authors.

## Funding

No external funding was received for this study.

## Ethics approval and consent to participate

Not applicable.

## Consent for publication

Not applicable.

## Declaration of Competing Interest

The authors declare that they have no known competing financial interests or personal relationships that could have appeared to influence the work reported in this paper.

## Data Availability

All data during study are included in this manuscript.
